# NeuroKinect: A Novel Low-Cost 3Dvideo-EEG System for Epileptic Seizure Motion Quantification

**DOI:** 10.1371/journal.pone.0145669

**Published:** 2016-01-22

**Authors:** João Paulo Silva Cunha, Hugo Miguel Pereira Choupina, Ana Patrícia Rocha, José Maria Fernandes, Felix Achilles, Anna Mira Loesch, Christian Vollmar, Elisabeth Hartl, Soheyl Noachtar

**Affiliations:** 1 Institute for Systems Engineering and Computers – Technology and Science (INESC TEC), and Faculty of Engineering (FEUP), University of Porto, Porto, Portugal; 2 Institute of Electronics and Informatics Engineering of Aveiro (IEETA), and Department of Electronics, Telecommunications and Informatics, University of Aveiro, Aveiro, Portugal; 3 Epilepsy Center, Department of Neurology, University of Munich, Munich, Germany; 4 Chair for Computer Aided Medical Procedures, Technische Universitat Munchen, Munich, Germany; Cleveland Clinic, UNITED STATES

## Abstract

Epilepsy is a common neurological disorder which affects 0.5–1% of the world population. Its diagnosis relies both on Electroencephalogram (EEG) findings and characteristic seizure−induced body movements − called seizure semiology. Thus, synchronous EEG and (2D)video recording systems (known as Video−EEG) are the most accurate tools for epilepsy diagnosis. Despite the establishment of several quantitative methods for EEG analysis, seizure semiology is still analyzed by visual inspection, based on epileptologists’ subjective interpretation of the movements of interest (MOIs) that occur during recorded seizures. In this contribution, we present NeuroKinect, a low-cost, easy to setup and operate solution for a novel 3Dvideo-EEG system. It is based on a RGB-D sensor (Microsoft Kinect camera) and performs 24/7 monitoring of an Epilepsy Monitoring Unit (EMU) bed. It does not require the attachment of any reflectors or sensors to the patient’s body and has a very low maintenance load. To evaluate its performance and usability, we mounted a state-of-the-art 6-camera motion-capture system and our low-cost solution over the same EMU bed. A comparative study of seizure-simulated MOIs showed an average correlation of the resulting 3D motion trajectories of 84.2%. Then, we used our system on the routine of an EMU and collected 9 different seizures where we could perform 3D kinematic analysis of 42 MOIs arising from the temporal (TLE) (n = 19) and extratemporal (ETE) brain regions (n = 23). The obtained results showed that movement displacement and movement extent discriminated both seizure MOI groups with statistically significant levels (mean = 0.15 m vs. 0.44 m, *p*<0.001; mean = 0.068 m^3^ vs. 0.14 m^3^, *p*<0.05, respectively). Furthermore, TLE MOIs were significantly shorter than ETE (mean = 23 seconds vs 35 seconds, *p*<0.01) and presented higher jerking levels (mean = 345 ms^−3^ vs 172 ms^−3^, *p*<0.05). Our newly implemented 3D approach is faster by 87.5% in extracting body motion trajectories when compared to a 2D frame by frame tracking procedure. We conclude that this new approach provides a more comfortable (both for patients and clinical professionals), simpler, faster and lower-cost procedure than previous approaches, therefore providing a reliable tool to quantitatively analyze MOI patterns of epileptic seizures in the routine of EMUs around the world. We hope this study encourages other EMUs to adopt similar approaches so that more quantitative information is used to improve epilepsy diagnosis.

## Introduction

Epilepsy is a common neurological disorder which affects 0.5–1% of the world population. The main symptom of epilepsy are epileptic seizures which typically occur unexpectedly. In between seizures most individuals with epilepsy function normally. Video-EEG monitoring is the most accurate tool for diagnosis and differential diagnosis of epilepsy. The seizure semiology and its relations to changes in the electroencephalogram (EEG) are the cornerstone of this diagnostic method [1].

Although quantitative methods for EEG analysis are established for many years, seizure semiology is still analyzed in most Epilepsy Monitoring Units (EMUs) by visual inspection, based on epileptologists’ subjective interpretation of the movements of interest (MOIs) that occur during recorded seizures.

Our group initiated attempts to quantify seizure movements in the last century with a 2D infrared (IR) marker-based approach [2] [3] [4] that was later used for several studies showing the clinical relevance of quantifying semiology of epileptic seizures [5] [6] [7] [8] [9]. Other groups used similar [10] 2D approaches reaching the same conclusions on its clinical relevance. For newborns, there were also some efforts in the literature that took advantage of the stereotypical limbs movements that seizures induce in these newborns and a relatively static posture they keep while in a neonate intensive-care unit (ICU) [11] [12].

The limitation of the 2D approaches were obvious: the difficulty of tracking the erratic MOIs, frequent marker occlusions and instability of the attached reflectors or sensors due to violent seizure associated movements. Eventually, the lack of precision when MOIs are not performed parallely to the image plane distorts the analysis. These limitations asked for a 3Dvideo-EEG system.

An evolution in this direction, based on the integration of a high-speed (200Hz) 6 infrared motion tracking camera system (Vicon plc., Oxford, UK) with a commercial 64/128 channels video-EEG system (XLTEK, London, ON, Canada [13]), has been reported elsewhere by our group [14]. We showed the advantages of using this technology in the routine of an EMU when compared to 2D approaches. The high cost of this system (≃ €100.000) and high maintainance demands (calibrations, reflectors placing) limits its widespread use in other EMUs around the world, especially in developing countries where funding is scarce.

Non-camera approaches for motion quantification in epileptic seizures have been presented relying on wearable inertial sensors such as gyroscopes, accelerometers or magnetometers. These approaches were used for seizure detection based on filtering [15] [16], principal component analysis [17] or artificial neural networks [18]. Although these systems rely on inexpensive hardware units, they need some maintenance (e.g. calibration, batteries, data synchrony, etc.) and need to be attached to the body in a way that does not detach with seizure-induced movement, not being suitable for all types of seizures, mainly the most violent ones.

Newly available RGB-D cameras (color and depth streams) such as the Microsoft Kinect [19], ASUS Xtion Pro Live [20] or Softkinect DS311 [21], have emerged from the gaming industry. They are promising enhancements in 3D computer vision approaches [22] and are available at very low-cost compared to previous similar cameras. They were already used for gait analysis showing sufficient accuracy when compared to the established and precise optical multi-camera motion capture system [23] [24]. Recently, a similar conclusion has been reached for patients with Parkinson’s disease [25] [26].

In this contribution, we present NeuroKinect—a low-cost, easy to setup and operate solution for a novel 3Dvideo-EEG system based on a RGB-D sensor (Microsoft Kinect) for 24/7 monitoring of a EMU bed. It does not require the attachment of any reflectors or sensors to the patient’s body and has a very low maintenance load, only associated to data management that can be largely automated. A comparative study of seizure-simulated MOIs using simultaneously the 6-camera Vicon system and the NeuroKinect is presented. Furthermore, we introduce the system 3D tracking algorithm that uses the RGB and depth data, and the corresponding kinematics analysis of 42 real seizure MOIs, 19 from temporal (TLE) and 23 from extratemporal (ETE) brain regions. We show that several quantified 3D parameters obtained with the developed system can discriminate TLE from ETE seizure MOIs considering a *p*<0.05 level. Finally, we compare the time needed to perform the presented kinematics analysis using our 3D tracking method vs. using a corresponding 2D frame by frame tracking procedure we have used in the past in similar studies [3] [4] [7] [27].

To the best of our knowledge, no similar low cost system is currently available with these characteristics and potential.

## Methods

### System architecture

**Fig 1:**
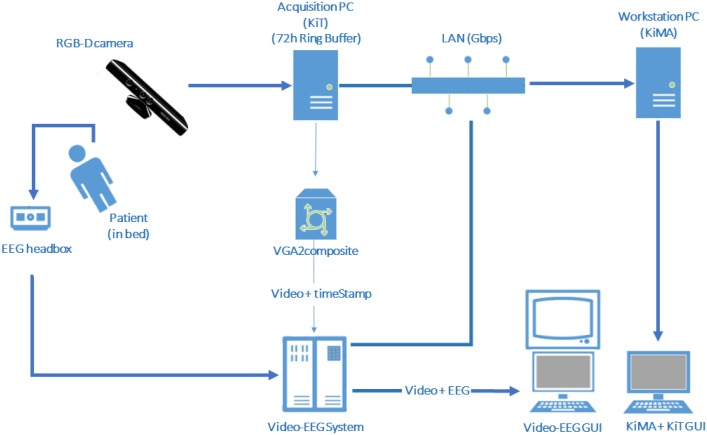
NeuroKinect architecture deployed inside the University of Munich EMU.

**Fig 2:**
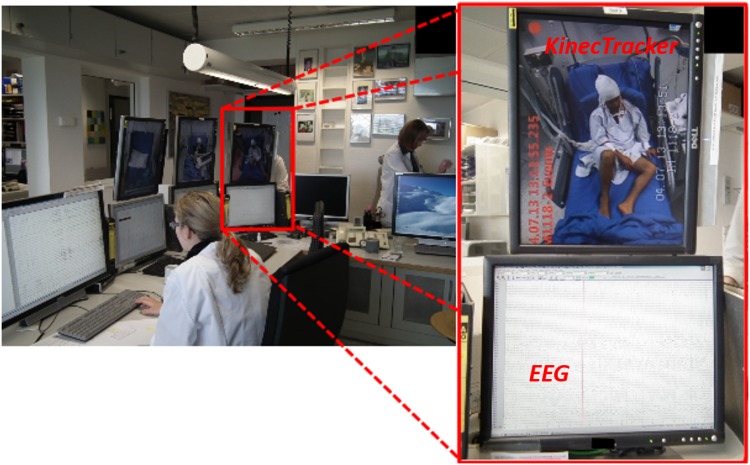
Aspects of the 3Dvideo-EEG system console installed in the routine of University of Munich EMU. Notice the KiT time-code is inserted in the video-EEG system for complete synchronization purposes. The system does not interferes with the EMU routine.

The archicheture of our 3Dvideo-EEG system can be depicted in Fig 1. It includes an RGB-D camera connected to an acquisition computer running KinecTracker (KiT), a custom developed software tool that handles all the 3D data acquisition workflow. The NeuroKinect is synchronized with the commercial video-EEG system through a VGA-to-composite video converter. The KiT screen data (that includes a msec time-stamp) are fed into the video-EEG system and integrated into the EMU routine operation console, as shown in Fig 2. Given the requirement of continuous 24/7 data acquisition and the heavy CPU load of data acquisition, a second dedicated computer (workstation PC) is connected through a high-speed Ethernet LAN so that data editing, storage and analysis can be performed using a second custom made software tool called KiMA (Kinect Motion Analyzer) while the system continues to acquire data 24/7.

**Fig 3:**
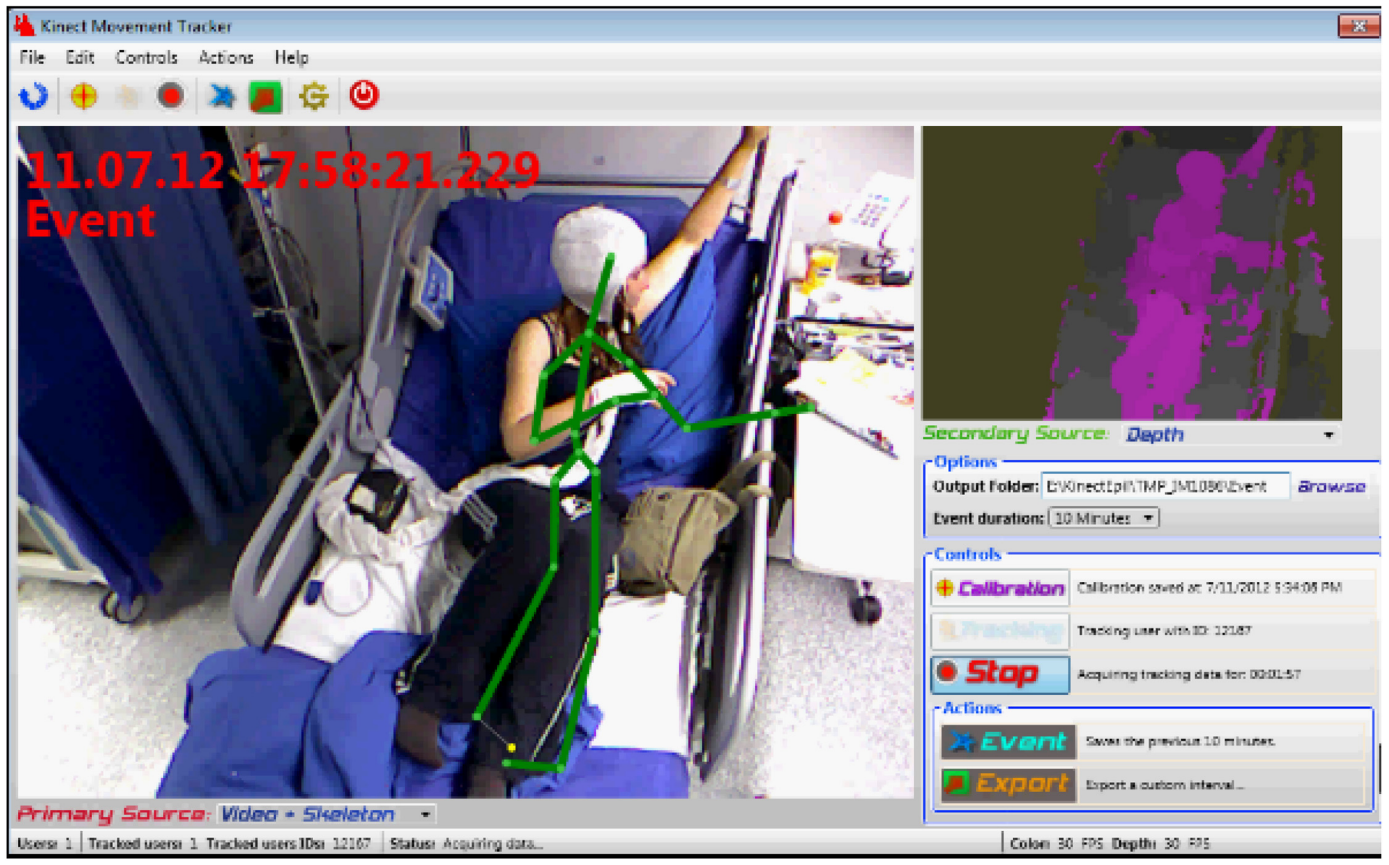
KiT first integration in the routine of University of Munich EMU (circa 2012).

The RGB-D camera (a Kinect v1 sensor, Microsoft Corp., Redmond, WA, USA) provides synchronized 640 × 480 8-bit color (RGB) and depth images at 30 frames-per-second (fps), and connects to the workstation PC through a USB port [22] generating a ± 2Gbytes/min data throughput. The depth information is obtained through a structured light 3D imaging technology based on an IR emitter and depth sensor. The NeuroKinect applications (KiT and KiMA) were developed in C# programming language using the Microsoft Kinect Software Development Kit (SDK) (version 1.5) [28]. It provides access to the raw data streams, including twenty 3D body joints data extracted by an embedded online tracking algorithm, given some assumptions in the processed scene as shown in the KiT graphical user interface (GUI) example depicted in Fig 3.

The KiT application enables the management of acquisition sessions performed with the system. Users can perform several actions such as calibration, start/stop data acquisition, insert a label associated to an given instant (e.g. beginning of a seizure), among other workflow controls. Furthermore, a ring-buffer solution for data acquisition was implemented to enable 24/7 continuous recording, required for a routine EMU within a hospital environment.

**Fig 4:**
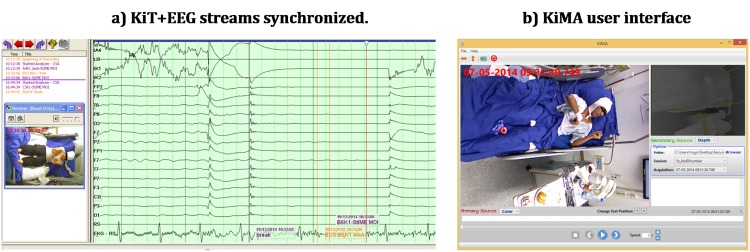
KiT+EEG and KiMA representations. Synchronized multiple EEG channels with the KiT software (Fig A). Optimized field-of-view of the Kinect installed in the epilepsy room, showed in KiMA’s user interface (Fig B). The typical distances range between the sensor and the bed center ranges from 1.9–2.1 meters.

Based on this ring-buffer solution, while KiT continuously acquires all data to this buffer, the user can mark the occurrence of Events (e.g. seizures) and within a “buffer size duration” (currently 72h but dependent on the buffer disk size), transfer the marked events to a database hosted by the workstation PC through the high-speed data connection using the KiMA tool (Fig 4). KiMA also enables editing transferred data files by marking new events or labeling specific moments, plug-in processing modules and export event data to run analysis pipelines using other tools (e.g. Matlab, Weka, etc.).

### Data acquisition

A comparative performance preliminary study between our low-cost system and an expensive multi-camera optical system was performed. NeuroKinect was installed over the same EMU bed where we have been using the Vicon system reported elsewhere [4]. A volunteer, expert in seizure semiology, was asked to perform a series of 10 different seizure-simulated MOIs while both 3D systems were acquiring data. 30 MOIs were tracked from trunk and left and right wrists body parts, following our previous findings on their importance to quantitative seizure semiology characterization on a 100 seizures 2D motion study [7].

Following this preliminary study, we proceeded to embed the NeuroKinect into the routine of the University of Munich EMU and established a data acquisition protocol with the technicians and epileptologists. The designed experimental protocol consists of several steps. Firstly, the scene background is saved before initiating the monitoring of a new patient. This image can be later used for background-subtraction purposes. Then the normal routine is carried on by putting the patient on the EMU bed, connecting the EEG electrodes, etc.

The system starts then running 24/7 storing data in the ring-buffer and waiting for a seizure to occur. As part of the EMU routine, seizures onsets are marked by the technicians in the video-EEG system and can be retrieved in the NeuroKinect. The EEG seizure pattern and its evolution is classified according to a system published earlier [29]. The ictal EEG is used for localization purposes and can be related to the 3D movement analysis at any given point in time (Fig 4A). Once the seizure is over, an event label can be inserted using the KiT GUI (Fig 3) triggering an email to the engineering team to take care of extracting the seizure data from the ring buffer. A large buffer allows several days of recording. This is particularly relevant when seizures occur during weekends. Based on KiT seizure labels, KiMA is used to cut and crop seizure data and store all data in the NeuroKinect database through the workstation PC. Patient monitoring using KiT and KiMA were authorized by the hospital’s Ethics Committee. The individuals in this manucrispt have given written informed consent (as outlined in PLOS consent form) to publish these case details.

### Camera position optimization

The preliminary study has shown that the built-in Kinect 3D body-joint position tracking algorithm [22] was not suitable for the complex EMU scene we were dealing with. That was already expected since it was developed for a “standing game” scene type and not a lying over bed body position. An example of such problems can be spotted in Fig 3, where the left arm of the patient is mistakenly tracked as the bed side. Given that finding, we optimized the camera position to obtain the best aspect ratio vs. resolution possible for the EMU scene. This was achieved by rotating the camera 90 degrees, as depicted in Fig 4B.

The dataset used in this study is composed of 42 seizure MOIs found in different parts of the patients’ body, 19 from TLE and 23 from ETE seizures distributed as depicted in Table 1.

**Table 1:** Information on 42 real seizures MOIs characteristics.

Sz Type	#Sz	#MOI	MOIs				#Frames	Duration (min)	Data size (Gb)
			Head	Left hand	Right hand	Left foot			
TLE	4	19	6	6	6	1	23,520	7.2	13.3
ETE	5	23	8	8	6	1	47,876	13.4	24.8
Total	9	42	14	14	12	2	71,396	20.6	38.1

These MOIs have a total of 71,396 frames (RGB and depth) covering over 20 minutes of ictal activity and totalizing almost 40 Gbytes of data to analyze.

### 3D semi-automatic tracking algorithm

Since we could not use the Microsoft Kinect built-in algorithm to track the 3D positions of the body joints, we developed a novel user-interactive algorithm to process the NeuroKinect data. This method joins the optical flow (OF) concept [30] [31] [32] applied to the 2D RGB data with depth segmentation over the 3^rd^ dimension enabled by the infrared radar depth images of the Kinect camera.

**Fig 5:**
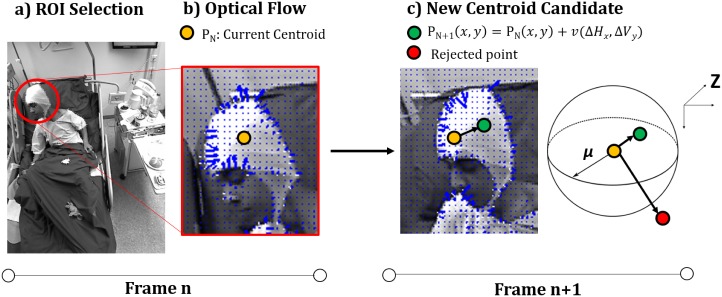
Schematic representation of the developed algorithm. The yellow and green colors are associated with the depth of the centroid in two consecutive frames. In frame n, the user selects and ellipsoid mask over the aimed ROI (Fig A). The resulting velocity vector is estimated (based on the OF pixel velocity vectors—in blue) and is used to calculate the next centroid (Fig B). In frame n + 1, the new centroid is calculated. If the depth of the estimated *P*_*n*+1_(*x*, *y*) is concordant with Eq 2, the estimation is accepted (Fig C).

The initial step of the tracking process consists in calculating the optical flow velocities over the RGB frames using the Horn-Schunk OF method [11] [12] [31]. Using the KiMA tool, the user can watch the seizure video and, through a plug-in module, visualize the corresponding overlap of the OF velocity vector fields to clearly identify the parts of the body where relevant seizure MOIs are present. The user is then interactively asked to select ellipsoid MOI masks that delimit the different parts of the body that should be tracked (e.g., head and left arm) and tracking is performed over the selected masks (see Fig 5A). The OF velocity vector fields within the defined mask (see Fig 5B) are used to estimate the next position of the mask with the following procedure:

Let *P*_*n*_(*x*, *y*) be the centroid of the ellipsoid mask in frame *n*. The centroid in frame *n*+1 is given by

$$\begin{array}{c} P_{n+1}\left(x,y\right)=P_{n}\left(x,y\right)+v\left(\Delta H_{x},\Delta V_{y}\right) \end{array}$$ (1)

where *v* stands for the OF velocity vector in both horizontal (*H*_*x*_) and vertical (*V*_*y*_) components.

Once the ellipsoid mask is defined, the median over the highest velocities vectors is calculated and then used to shift the centroid in the calculated proportion. The median was considered after performing the Jarque-Bera test [33] over 10 MOIs and confirming that the output values obtained did not follow a normal distribution. Furthermore, the possibility to select a certain percentage of the highest velocities was inserted so that it enables a filtering feature to the tracking noise. After the estimation of the next mask position, a depth criteria - Eq (2) - is applied in order to auto-correct the estimated next position and minimize tracking errors through the following procedure:

Let *Z*_*n*_ and *Z*_*n*+1_ be the depth values associated with the points (*x*_*n*_, *y*_*n*_) and (*x*_*n*+1_, *y*_*n*+1_) in frames *n* and *n* + 1, respectively. Once the new centroid is calculated, the following criteria is applied:

$$\begin{array}{c} \left\{ \begin{array}{c} |Z_{n}\left(x_{n},y_{n}\right)-Z_{n+1}\left(x_{n+1},y_{n+1}\right)|\leq \alpha \Rightarrow \text{Accepted}\\ |Z_{n}\left(x_{n},y_{n}\right)-Z_{n+1}\left(x_{n+1},y_{n+1}\right)|>\alpha \Rightarrow \text{Rejected} \end{array}\right. \end{array}$$ (2)

So, if the shifted mask centroid is within a certain depth range (*α*—set by the user) when compared to the previous position, the centroid is labeled as valid and the mask is shifted automatically as defined by Eq (1).

If the calculated centroid is rejected, the algorithm searches for the nearest neighbor inside the ellipsoid ROI that fulfill criteria Eq (2), given by

$$\begin{array}{c} \sqrt{{\left(x_{n+1}-x_{n}\right)}^{2}+{\left(y_{n+1}-y_{n}\right)}^{2}}\leq \mu \end{array}$$ (3)

where *μ* represents a fixed distance nearby the previous centroid, a point that satisfies Eq (2) and continues its tracking to the next frame. Finally, in case this auto-correction process is unable to find a centroid within the depth criteria, the user is requested to interactively re-position the centroid of the mask.

The process is repeated until all frames are analyzed. A schematic representation of this process is depicted in Fig 5.

Finally, a Savitzky-Golay FIR filter [34] is applied to the MOI sequences for smoothing purposes.

### Quantified metrics

Once movement tracking is completed, 3D quantification is performed by feeding the MOIs tracking time series (*X*_*i*_, *Y*_*i*_, *Z*_*i*_) into a custom made Matlab program that reads KiMA exported files. 3D quantification is performed after mapping the resulting 2D tracking information to the 3D Kinect coordinate system, using the available mapping method [35].

For all MOIs we computed a set of 56 + 23 quantified metrics and studied their significance level in discriminating the two types of seizure MOIs of the dataset. We start by computing the maximum, median, mean, minimum and standard deviation (std) of velocity, acceleration and jerk (second derivative of velocity) [36]. Movement displacement (MD—euclidean distance between the MOI initial and final positions) and the covered distance (CD—sum of the euclidean distances between MOI consecutive positions) per MOI were also calculated.

**Fig 6:**
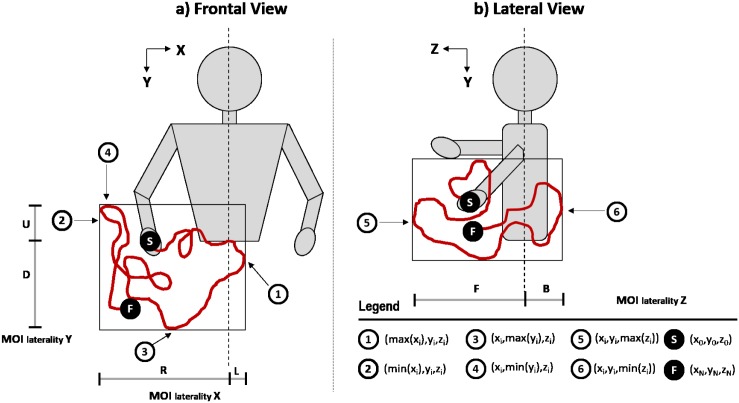
3D movement extent (ME) analysis and movement laterality of a seizure MOI. The solid line inside each rounding box represents movement extent, calculated as the maximum volume traveled by the MOI and limited by the three-dimensional maxima 1, 2, 3, 4, 5 and 6. The dotted line represents the medial planes, used as reference to calculate the movement laterality in each direction (R-right, L-left, F-front, B-back). In this example, a mostly right, down and front laterality MOI is represented.

To these 17 measures, we added a new parameter—3D movement extent (ME)–that is a generalization for 3D of a previous 2D measure we have been using for our 2D studies [7]. A graphical representation of this parameter is provided in Fig 6. We also calculated statistical values regarding the movement laterality per axis (X, Y and Z).

Laterality is the maximum distance of a MOI trajectory in relation to the median planes (XY and YZ)—left/right; down/up and back/front, respectively. A criterion was also applied to assess whether there was a clear tendency of the MOI to travel in a certain direction. In such case, the distance between the MOI position and the medial plane is calculated (Fig 6).

Finally, we introduce several new seizure quantification parameters—Seizure Movement Extent (SME); Seizure Covered Distance (SCD); Seizure Movement Displacement (SMD); Seizure Aggregate Velocity/Acceleration/Jerk (SAV, SAA, SAJ), which correspond to 18 seizure quantification parameters. SME is the aggregation of the MEs of all MOIs involved on a given seizure. For example, if a seizure presents hand automatisms and head rotations, these parameters aggregates the ME of both hands’ MOIs and the head MOI resulting in the overall volume covered by that seizure MOIs, as defined in Eq (4):

$$\begin{array}{c} SME\left(s\right)=\sum_{i=1}^{m}MEs\left(s,i\right) \end{array}$$ (4)

where *s* stands for seizure and *m* represents the number of MOIs in that seizure. The same analogy is applied for the remaining variables.

Given the dataset characteristics (Table 1) we tested the hypothesis that one or more of our resulting quantified metrics would significantly discriminate temporal from extratemporal seizure MOIs. The duration of the seizures and seizure patterns (set of MOIs present in a seizure) were also added to the discrimination tests.

### Statistical Analysis

All 56 + 23 seizure quantitative parameters for the two different seizure pattern groups (temporal vs. extratemporal) were compared using the Wilcoxon signed rank test [37]. In addition, Fisher’s exact test [38] was also performed to evaluate possible significant differences between labeled lateral MOIs. Differences were considered statistically significant at *p*<0.05.

## Results

### NeuroKinect vs Vicon: A comparative preliminary study

**Fig 7:**
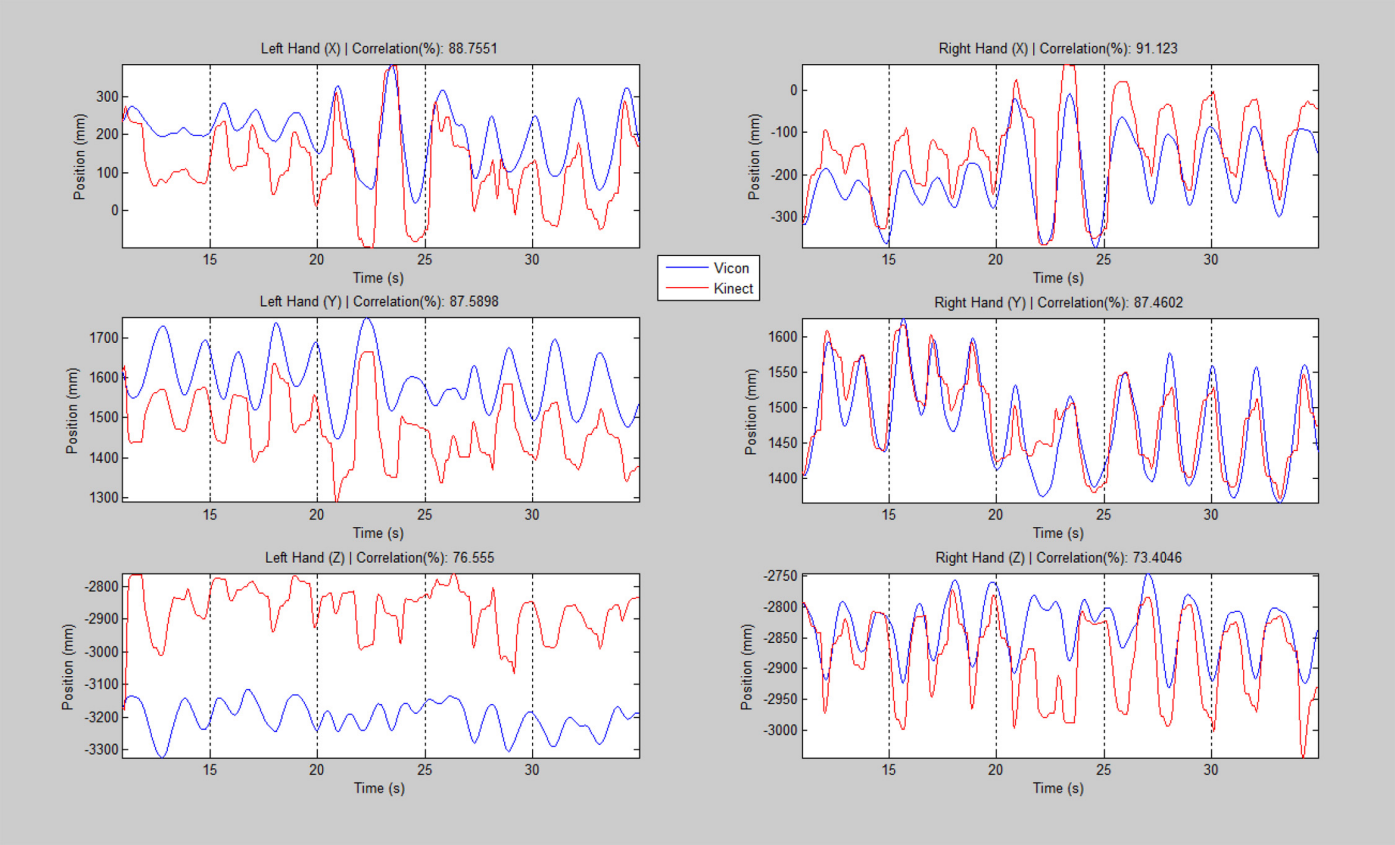
Correlation between Vicon and the NeuroKinect System. The average correlation for all 3 axes as 84.2%.

MOIs were acquired at 200 Hz by the Vicon system and 20 Hz by KiT. To perform a comparison, the Vicon motion signals were down-sampled to 20 Hz. For all stimulated MOIs, signals were low-pass filtered and then compared visually and through a 3D correlation analysis using a Matlab custom-made program. The overall average correlation was 84.2% considering all 3 axes. Details of this study can be found in Fig 7 and in S1 Appendix.

### 3D seizure data analysis

The results of the performed statistical analysis are summarized from Tables 2–4. The obtained results showed that TLE MOIs were significantly shorter than ETE (mean = 23 seconds vs 35 seconds, *p*<0.01). Significant differences were also found in the MOIs jerk, indicating that TLEs presented higher jerking than ETEs (mean = 345 ms^−3^ vs 172 ms^−3^, *p*<0.05). Movement displacement also separated both seizure MOI groups (mean = 0.15 m vs 0.44 m, *p*<0.001).

**Table 2:** MOI parameters with highest p-score from patient’s 42 seizures MOIs.

Sz Type	#MOIs	Velocity (ms^−1^)		Acceleration (ms^−2^)		Jerk (ms^−3^)		MD (m)	CD (m)	ME (m^3^)	MOI duration (s)
		Median	Sth	Mean	Sth	Mean	Sth				
TLE	19	0.086 ± 0.064	0.58 ± 0.41	12 ± 8.4	38 ± 33	345 ± 287	1204 ± 1151	0.15 ± 0.11	6.3 ± 4.2	0.068 ± 0.11	23 ± 6.6
ETE	23	0.068 ± 0.063	0.46 ± 0.29	8.6 ± 4.6	23 ± 17	172 ± 127	574 ± 506	0.44 ± 0.29	7.6 ± 2.9	0.14 ± 0.16	35 ± 10
*p*	n.s.	n.s.	n.s.	n.s.	<0.05	n.s.	<0.001	n.s.	<0.05	<0.01

[Note.] Values are given as mean ± std. n.a.: not applicable. n.s.: not significant.

**Table 3:** MOI laterality parameters with highest p-score.

Sz Type	#MOIs (L-R)	MOI laterality X		#MOIs (D-U)	MOI laterality Y		#MOIs (B-F)	MOI laterality Z	
		Left (cm)	Right (cm)		Down (cm)	Up (cm)		Back (cm)	Front (cm)
TLE	14 (6–8)	1.5 ± 0.8	1.0 ± 0.89	13 (8–5)	0.35 ± 0.4	0.53 ± 0.075	14 (8–6)	5.4 ± 3.9	10 ± 7.9
ETE	19 (7–12)	6.1 ± 8.6	0.88 ± 0.87	18 (12–6)	1.1 ± 0.14	1.8 ± 0.25	19 (12–7)	2.2 ± 1.0	3.2 ± 2.3
*p*	n.a.	n. s.	<0.05	n.a.	<0.001	n. s.	n.a.	n. s.	<0.001

[Note.] Values are given as mean ± std. n.a.: not applicable. n.s.: not significant

**Table 4:** Seizure parameters with highest p-score.

Sz Type	#Sz	SAV (ms^−1^)	SAA (ms^−2^)	SAJ (ms^−3^)	SME (m^3^)	SMD (m)	SCD (m)	Sz duration (s)
		Max	Max	Max				
TLE	4	0.332 ± 0.217	26.3 ± 23.9	940.9 ± 960.2	0.32 ± 0.25	0.69 ± 0.57	30.1 ± 19.2	35 ± 3
ETE	5	0.261 ± 0.257	15.8 ± 15.2	509.1 ± 501.5	0.55 ± 0.79	1.9 ± 2.1	32.3 ± 21.0	55 ± 30
*p*	n.a.	n.s	n.s	n.s	n.s	n.s	n.s	n.s

[Note.] Values are given as mean ± std. n.a.: not applicable. n.s.: not significant

Additionally, the analysis of the movement extent separated TLE MOIs from ETE MOIs, as it was greater in the extratemporal than in the temporal seizures (mean = 0.14 vs 0.068 m^3^ m^3^, *p*<0.05).

In terms of movement laterality, highly significant differences were found in terms of the movements characteristics, as it can be depicted in Table 3. ETE MOIs showed a trend of reaching further distances in both X and Y directions, which is also confirmed by the volume occupied by these MOIs. It can also be perceived from Table 3 that not all MOIs had a preferred movement direction; for example, for the movements along the X axis, from the 19 temporal MOIs, only 14 had a clear movement direction, and from those 14, six were towards the left direction and the remaining in the opposite (right) direction.

Based on the performed classification, we then evaluated whether there were any significant differences between TLE and ETE movements in the same direction. It can be seen (Table 3) that there are discriminative differences between movements in both X (right; std = 0.001 m vs 0.0088 m, *p*<0.05) and Y (down; std = 0.0035 m vs 0.011 m, *p*<0.001) direction, but also in the Z axis, namely in the labeled front movements (front; maximum = 0.1 m vs 0.032 m, *p*<0.001).

We also evaluated whether there is any significant asymmetry between ictal movements of TLE and ETE seizures. For that purpose, three 2x2 contingency tables were designed (using the information available in Table 3 from the number of MOIs) and the Fisher’s exact test was performed, with the result being a non-discriminative p-value (*p* > 0.05).

Finally, considering the seizure quantification parameters, presented in Table 4, no ETE seizure had faster movements than TLE. The duration of the ETE seizures (55 ± 30 seconds vs 35 ± 3 seconds, *p* > 0.05) showed a trend of being longer than that of the TLE seizures. Also, the same trend is seen for the seizure movement extent, movement displacement and covered distance (*p* > 0.05).

### 3D Tracking Algorithm Performance

Comparing our semi-automatic approach with a state-of-the-art 2D motion analysis software, MaxTRAQ [39], that we have used previously, it not only provides 3D information but also allows saving a considerable amount of time by avoiding the manual labeling of every centroid for each frame. We verified that, when using our approach, the user would only make four interactions (assuming 4 joints being tracked) in 1 out of 10 frames, which would take 20 seconds. Conversely, using MaxTRAQ it is necessary to perform four interactions per frame (i.e. forty interactions per 10 frames). Assuming that an experienced user spends roughly 4 s per interaction using MaxTRAQ, the user would save at least 140 seconds per set of 10 frames. Considering a seizure with the duration of 60 s (1800 frames), then it is possible to save a minimum of 420 minutes per analyzed seizure. With our dataset, we estimate that we saved 140 hours in its analysis (87.5% less time) using our approach than if we used a 2D approach.

## Discussion

### Novel 3Dvideo-EEG system based on a RGB-D camera

This feasibility study shows that our low cost 3D movement analysis system is easily usable in the clinical routine of a standard EMU setting and is capable to track and identify sets of MOIs which allow the discrimination between seizures arising from temporal and extratemporal brain areas. This system allows to overcome major limitations of our previous 2D marker-based system using a monochrome camera and infrared markers attached to the patient’s body [3] [4].

The direct comparison to a high cost multicamera system [14] shows that our low cost markerless system provides reliable 3D data. A challenge of the system is that one has to deal with a high data volume due to long-term monitoring. This was addressed by using a ring-buffer concept that facilitates continuous monitoring of a given patient. Both KinecTracker and KiMA applications were designed in a way that would ease the physician’s interaction with the software.

The parallel use of a RGB-D camera with a conventional video-EEG recording allows full synchronization of the signals. This is particularly important for the evaluation of ictal EEG localization with regard to seizure related movements. It is well known that spread of epileptic activity over the brain leads to changes of characteristic seizure associated movements [40].

### 3D MOIs seizure analysis

Analysis of epileptic seizure semiology relies basically on qualitative criteria which makes it prone to inter-observer discrepancy [41]. It has been shown in several studies of our group that even 2D quantitative movement analysis provides valuable objective information on the localization of the epileptogenic zone. This has been documented for head version during epileptic seizures [5] [7] and epileptic automatisms [6] [9]. The present system overcomes the limitation of the 2D analysis and will allow more accurate movement quantification.

Our 3D analysis shows that TLE seizure related movements are faster and more confined in space than ETE. On the other hand, the ETE patterns are longer and cover a higher tri-dimensional volume. The ETE movement occurs at lower velocities and is progressive in space. For the purpose of this paper, we focused on the statistical analysis of the above mentioned movement parameters. However, other approaches would also be of interest such as pattern recognition and machine learning. MOI pattern analysis did not reach statistical significance to discriminate TLE and ETE seizures in this study, probably due to the low number of seizures included in this pilot feasibility study.

### Kinect for Windows v2 sensor

Kinect for Windows Developer Program Preview [42] enabled research groups and developers to be provided with the alpha version of the new Kinect sensor which has been recently released to the market. BRAIN (Biomedical Research and INnovation group (http://brain.inesctec.pt/) was one of the first 500 groups being granted with this new sensor, in early 2014. The sensor has been re-engineered with wider field-of-view, higher resolution of the color data (1920 × 1080 High-Definition), higher depth fidelity due to the use of time-of-flight technology, more stable body tracking algorithm and overall SDK improvements [43].

**Fig 8:**
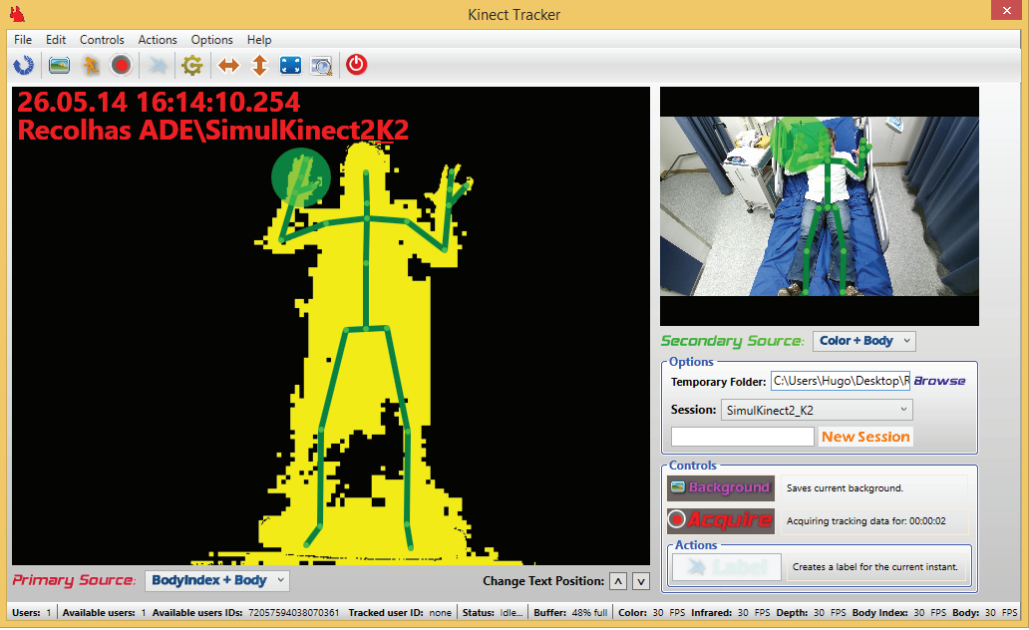
KiT v2 from LMU Bed: Trial tests to assess the new sensor robustness. In the primary and secondary source, the new color and bodyindex stream, as well as the 3D joints estimations of the human body are presented.

Kinect v2 sensor was recently integrated into the 3Dvideo-EEG system. From the acquisitions carried out so far, Kinect v2 seems to provide better information than the Kinect v1 sensor used in the present study, as it can be seen in Fig 8 and in S2 Appendix.

## Conclusion and Future Work

In this contribution, we introduce a novel 3D-video-EEG system, based on a low-cost markerless motion capture system that has been integrated into a routine EMU setting, allowing 24/7 patient monitoring. The system has been in operation for approximately one year and is producing valuable data every day now. To our knowledge, we are gathering the only 3D-video-EEG database in the world used in a routine clinical epileptology department. The present paper is a report of the first version of this system and the first dataset extracted form our multimedia clinical database, approaching the methods behind the acquisition and processing of seizure semiology. A new version of the system is already producing data which is now equipped with the new Kinect v2 sensor. It is providing more accurate information than the previous sensor, and we are also able to relate the quantified motion information here reported with EEG events occurring in the course of the seizure. Automation methods are not covered in this paper but we intend to do so in the coming publications. Seizure types or psychogenic vs. real seizure separation and seizure automated detection are in the horizon of the next challenges we are already tackling.

We also believe that the low-cost aspect of the system has the potential and the strengths to be spread and deployed in the routine of multiple epilepsy units around the world. We are open to receive requests from other EMUs around the world so that our development is used for the benefit of the larger number of patients possible.

To the best of the authors’ knowledge, the results here reported constitute the first usage of a Kinect sensor (both v1 and v2) in a real hospital environment, in the context of seizure semiology analysis.

## Supporting Information

S1 Appendix Correlation between Vicon and the NeuroKinect System. (PDF)

S2 Appendix KiT v2 from LMU Bed: Trial tests to assess the new sensor robustness. (MP4)
