# Supplementary material for: NeuroKinect: A Novel Low-Cost 3Dvideo-EEG System for Epileptic Seizure Motion Quantification
Source: PLoS One. 2016 Jan 22;11(1):e0145669. doi: 10.1371/journal.pone.0145669 (PMC4723069; doi:10.1371/journal.pone.0145669)
Supplement: S1 Appendix — (PDF) [file pone.0145669.s001.pdf]

NeuroKinect: A novel low-cost 3Dvideo-EEG system for epileptic seizure motion quantification

S14- Report 1 - Correlation between Vicon and the NeuroKinect System.

A comparative performance preliminary study between our low-cost system and the expensive multi-camera optical system (Vicon) was performed.

A volunteer, expert in seizure semiology, was asked to perform a series of 10 different seizure-simulated MOIs while both 3D systems were acquiring data. 30 MOIs were tracked from trunk and left and right wrists body parts.

MOIs were acquired at 200 Hz by the Vicon system and 20 Hz by KiT. To perform a comparison, the Vicon motion signals were down-sampled to 20 Hz. For all stimulated MOIs, signals were low-pass filtered and then compared visually and through a 3D correlation analysis using a Matlab custom-made program.

Figure 1 presents the correlation obtained on the relative position between the Kinect (in red) and the Vicon (in blue) system. The overall average correlation was 84.2% for all 3 axes.

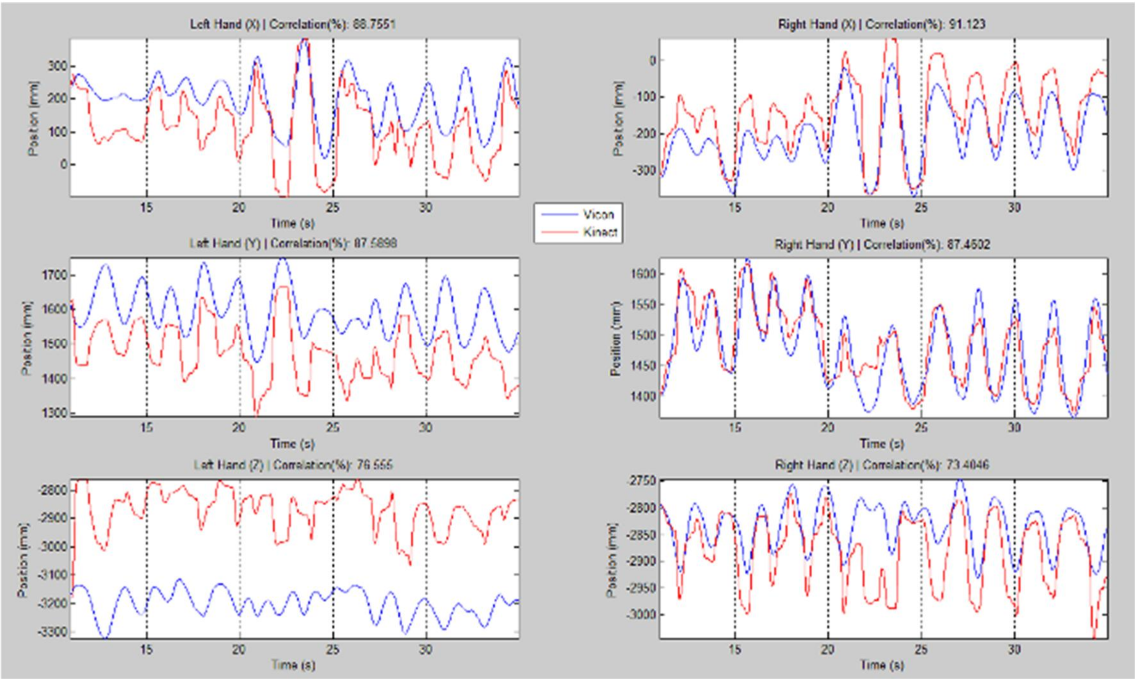

Figure 1 - Correlation between Vicon and the NeuroKinect System. The average correlation for all 3 axes as 84.2%
